# UHPLC-MS/MS method for analysis of sobuzoxane, its active form ICRF-154 and metabolite EDTA-diamide and its application to bioactivation study

**DOI:** 10.1038/s41598-019-40928-5

**Published:** 2019-03-14

**Authors:** Petra Reimerová, Anna Jirkovská, Hana Bavlovič Piskáčková, Galina Karabanovich, Jaroslav Roh, Tomáš Šimůnek, Petra Štěrbová-Kovaříková

**Affiliations:** Faculty of Pharmacy in Hradec Králové, Charles University, Akademika Heyrovského 1203, 500 05 Hradec Králové, Czech Republic

## Abstract

Sobuzoxane (MST-16) is an approved anticancer agent, a pro-drug of bisdioxopiperazine analog ICRF-154. Due to the structural similarity of ICRF-154 to dexrazoxane (ICRF-187), MST-16 deserves attention as a cardioprotective drug. This study presents for the first time UHPLC-MS/MS assay of MST-16, ICRF-154 and its metabolite (EDTA-diamide) in cell culture medium, buffer, plasma and cardiac cells and provides data on MST-16 bioactivation under conditions relevant to investigation of cardioprotection of this drug. The analysis of these compounds that differ considerably in their lipophilicity was achieved on the Zorbax SB-Aq column using a mixture of aqueous ammonium formate and methanol as a mobile phase. The biological samples were either diluted or precipitated with methanol, which was followed by acidification for the assay of MST-16. The method was validated for determination of all compounds in the biological materials. The application of the method for analysis of samples from *in vitro* experiments provided important findings, namely, that (1) MST-16 is quickly decomposed in biological environments, (2) the cardiac cells actively metabolize MST-16, and (3) MST-16 readily penetrates into the cardiac cells and is converted into ICRF-154 and EDTA-diamide. These data are useful for the in-depth examination of the cardioprotective potential of this drug.

## Introduction

Bisdioxopiperazines are effective anticancer agents; they are inhibitors of topoisomerase II (TOP II), an enzyme that manages conformational changes in DNA topology and is essential for DNA replication and RNA transcription^1^. However, poor solubility in aqueous environments and low bioavailability after oral administration significantly limits their potential for clinical use^2^. To overcome this issue, more water-soluble pro-drugs that are activated to the original bisdioxopiperazines were synthesized^1^.

**Figure 1:**
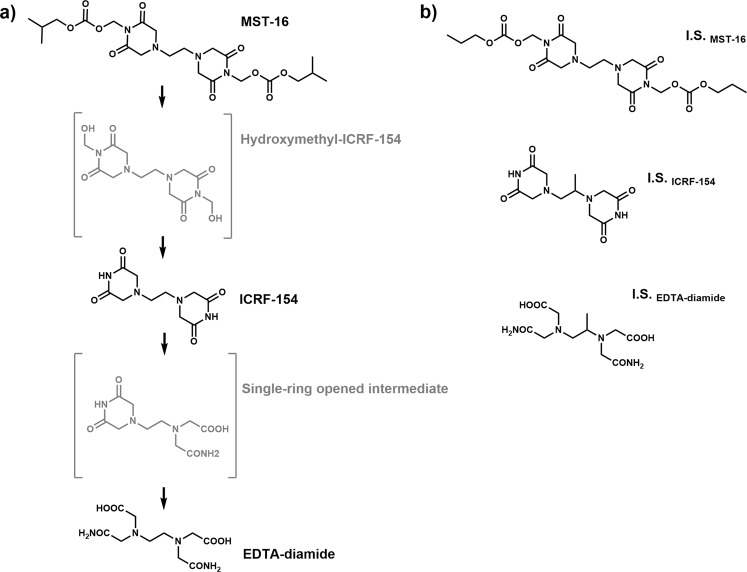
**(a)** The proposed activation of MST-16 to ICRF-154 and EDTA-diamide and **(b)** the chemical structures of the internal standards, I.S._MST-16_, I.S._ICRF-154_ (racemic form of dexrazoxane), and I.S._EDTA-diamide_(ADR-925). The suggested intermediates of MST-16 activation that were not detected in this study are shown in parenthesis.

Sobuzoxane (MST-16, Fig. 1a) is the first pro-drug of this group approved for clinical use as an anticancer drug in Japan^2,3^. MST-16 is supposed to be activated by the hydrolysis of ester bond to hydroxymethyl-ICRF-154^4^ that is followed by release of formaldehyde and the formation of the active compound, ICRF-154^5^ (Fig. 1a). The anticancer activity of MST-16 is attributed to TOP II inhibition by ICRF-154^6^, albeit it still remains unclear whether the intact pro-drug can also play a role.

Although bisdioxopiperazines have been developed primarily as antitumor agents, it was demonstrated during their preclinical development that they are able to protect the heart against anthracycline-induced toxicity, and dexrazoxane (ICRF-187, DEX, Fig. 1b) has been approved for clinical use as a cardioprotective agent^7–9^. Despite the long history of DEX in clinical practice, the mechanism responsible for its cardioprotective effect has not yet been completely explained. For decades, the effect has been ascribed to the iron-chelating activity of its active metabolite, ADR-925 (Fig. 1b)^9,10^. Recently, it was demonstrated that the parent DEX may instead protect the heart by catalytic inhibition of the beta isoform of TOP II^11–14^.

Scarce data on the cardioprotection of MST-16 have been available so far, but they suggest a high potential for this pro-drug^4,15,16^. Due to the high structural similarity of its active form ICRF-154 to DEX along with the ability of ICRF-154 to interact with TOP II^6^, the compound deserves a thorough assessment of its cardioprotective potential. Furthermore, data on the possible role of bioactivation and metabolism of MST-16/ICRF-154 may contribute to understanding the mechanism(s) responsible for cardioprotection in the bisdioxopiperazines group. This is important for further development of novel cardioprotective drugs.

Although the metabolism of ICRF-154 has not been studied yet, this compound is expected to undergo gradual hydrolytic opening of the bisdioxopiperazine rings similarly as it has been previously described in DEX^9^. This should yield a single-ring opened intermediate metabolite and subsequently, in the next step, an EDTA-like chelating compound, EDTA-diamide (Fig. 1a). A proper bioanalytical method for the simultaneous determination of MST-16, ICRF-154 and the EDTA-diamide metabolite in relevant biological materials is a basic methodological tool for examination of the pro-drug’s activation and metabolism. The method is also a prerequisite for the investigation of the relevance of MST-16/ICRF-154 metabolism to their prospective cardioprotective effects. Although MST-16 has been used in clinical practice since 1994, there are still very sporadic reports available in scientific databases on its bioanalysis. The only previously published method is the HPLC-UV assay of ICRF-154 in plasma after administration of MST-16 to rats. However, this assay did not allow for simultaneous determination of ICRF-154 with either the pro-drug compound, MST-16 or the prospective metabolite, EDTA-diamide. There are also no data on validation parameters^5^. The limited attention paid to this analysis may be caused by the complications associated with the chromatographic assay of these compounds. The simultaneous analysis of compounds of very distinct polarities such as MST-16, ICRF-154 and the metabolite usually requires a relatively long analysis time when using common C18 columns. Furthermore, the high polarity of EDTA-diamide leading to poor retention on the column, and the iron chelation ability of this compound may result in deterioration of the peak shape, poor repeatability of injection and loss of sensitivity^17^. As a consequence, there are also scarce data regarding MST-16 stability and activation in biological materials.

In this study, we aimed to develop and validate the first UHPLC-MS/MS method for simultaneous analysis of MST-16, ICRF-154 and EDTA-diamide in cell culture medium, buffer, rat neonatal ventricular cardiomyocytes (NVCM) and plasma. The method was further applied to a comprehensive *in vitro* study to obtain the first data on MST-16 bioactivation/stability in biological materials under conditions that are relevant for investigation of its cardioprotection.

## Results and Discussion

### UHPLC-MS/MS method development and validation

Bioactivation of MST-16 as well as further metabolism of its active compound - ICRF-154 probably involves series of hydrolytic reactions that may comprise formation of several intermediate metabolites^4,9^. In this study we focused on development of the assay for analysis of the parent pro-drug (MST-16), its main active form (ICRF-154) and expected endpoint metabolite (EDTA-diamide). The UHPLC-MS/MS method was selected for simultaneous assay of these compounds as this technique provides high selectivity, sensitivity and the potential for high-throughput analysis. Moreover, when using in appropriate settings, it allows for robust, accurate and precise assay of the trace amount of drugs in a complex biological matrix^18^. Apart from advantages, the simultaneous UHPLC-MS/MS analysis of MST-16, ICRF-154 and EDTA-diamide is complicated mainly by the poor retention of a highly polar metabolite on a reversed-phase column along with the distinct polarities of the analytes. These problems usually resulted in the need for a relatively long run time. The analysis of DEX and its analog (JR-311) along with the corresponding polar metabolite required 30 and 20 minutes, respectively^19,20^. Although in this study we also needed to analyze the highly lipophilic pro-drug together with ICRF-154 and the hydrophilic metabolite, one of the main priorities was to reduce the run time of the chromatographic analysis. The stationary phases tested herein  were selected based on our two previous findings: (1) common C18 columns were not able to retain highly polar EDTA analogs and (2)  hydrophilic-interaction chromatography (HILIC), although it may improve retention of highly polar metabolites, is not suitable for analysis of a more lipophilic active compounds^20,21^.

Hence, we tested three reversed stationary phases with different chemistries that are suitable for the analysis of polar compounds. Although porous graphitic carbon (Hypercarb) provided retention of EDTA-diamide, the lipophilic MST-16 was strongly retained on the column which resulted in a long analysis time and the deterioration of its peak shape. Synergi Polar, a column used in our previous study for analysis of DEX^20^, and Zorbax SB-Aq provided comparable results in terms of retention of EDTA-diamide and ICRF-154, however the latter allowed for an improved peak shape of MST-16. Moreover, the first column is not commercially available with sub 2 µm particles for UHPLC. Therefore, Zorbax SB-Aq column was finally selected as the appropriate stationary phase. The chelation ability of the analytes is known to complicate the chromatography due to artificial formation of complexes with trace ions in a chromatographic system that resulted in poor peak shape and loss of sensitivity. This can be successfully overcome by addition of EDTA or another chelator to the mobile phase^17,22,23^. However, these nonvolatile additives can decrease the sensitivity of MS detection. In the case of analysis of EDTA-diamide, we found that flushing the column with EDTA solution prior to the first use is sufficient to reach adequate chromatographic performance^23^. The mobile phase composition  and  the gradient profile, were optimized to achieve simultaneous analysis of all analytes with sufficient sensitivity, column performance and acceptable run time. Ammonium formate as a mobile phase additive enhanced detector response for EDTA-diamide, which was a critical analyte in terms of sensitivity. In contrast, any presence of formic acid (even 0.1%) led to signal suppression for EDTA-diamide. Unlike acetonitrile, methanol in the mobile phase was beneficial in terms of sensitivity. Due to the distinct physical/chemical properties of the analytes, three internal standards (I.S.) were selected, which were structurally close analogs for each of the analytes (Fig. 1b). Quantification was done using selected reaction monitoring (SRM) to maximize selectivity. For each analyte and its I.S., one transition was repeated 5 times which allowed for a 2x increase in sensitivity. This can be explained by the fact that the summation of the signal obtained by repeated SRM transitions is greater that the summation of the random noise. This phenomenon was also described by Pauwels *et al*.^24,25^.

**Figure 2:**
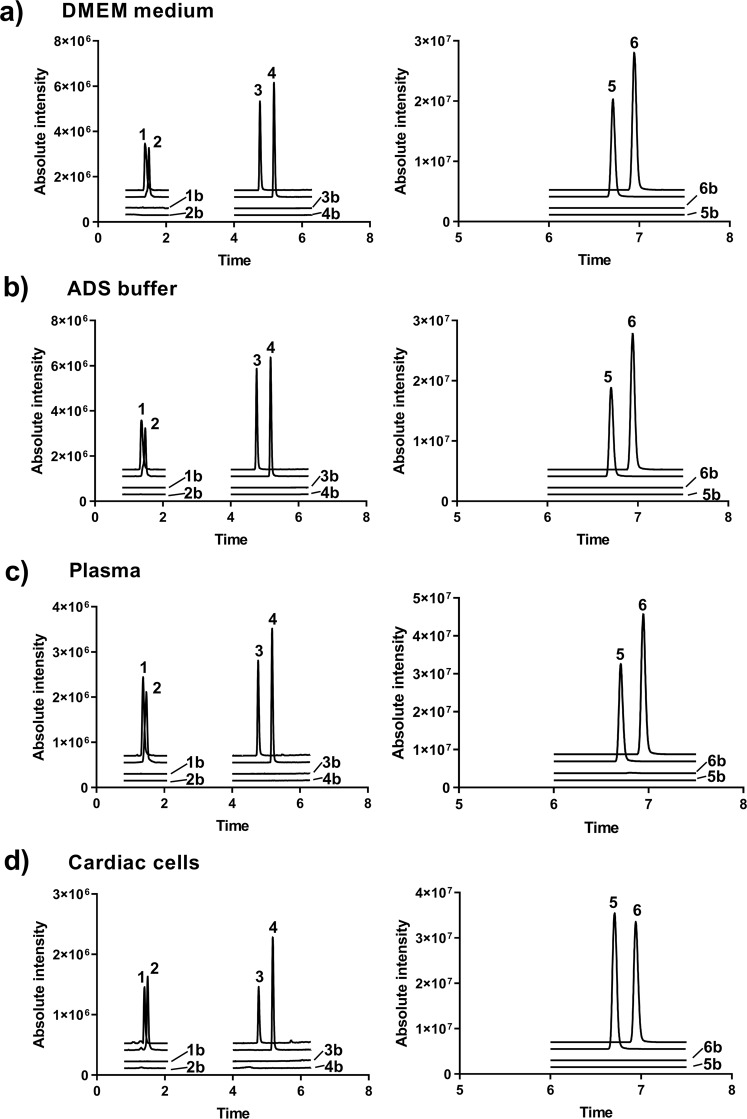
Representative chromatograms of UHPLC-MS/MS analysis of QC samples. **(a)** the medium (DMEM), **(b)** the  buffer (ADS) and **(c)** plasma spiked with EDTA-diamide, ICRF-154, MST-16, internal standards (50 µM), and the corresponding blank samples. **(d)** Cardiac cell samples spiked with EDTA-diamide, ICRF-154, MST-16 (30 pmol/10^6^ cells), internal standards (50 pmol/10^6^ cells), and corresponding blanks. (1) EDTA-diamide, (2) I.S._EDTA-diamide_, (3) ICRF-154, (4) I.S._ICRF-154_, (5) I.S._MST-16_, (6) MST-16. (1b) blank of EDTA-diamide, (2b) blank of I.S._EDTA-diamide_, (3b) blank of ICRF-154, (4b) blank of I.S._ICRF-154_, (5b) blank I.S._MST-16_, (6b) blank of MST-16. The chromatograms were monitored in SRM. (Details are specified in Table 6).

Apart from separation, the distinct polarities of the analytes complicate the use of a single sample treatment procedure for all compounds. Furthermore, a fast sample clean-up method was of particular importance, due to the suspected rapid conversion of MST-16 to ICRF-154 in biological materials. Hence, simple protein precipitation with ice-cold methanol was chosen for plasma and NVCM samples, which provided acceptable recovery of all analytes and required a short time. The same solvent mixed with water (20:80, *v/v*) was also used for dilution of Dulbecco’s Modified Eagle’s medium (DMEM) and buffered saline (ADS buffer, for exact composition see Chemicals and material section) samples prior to analysis. To increase the post-preparative stability of MST-16, the samples used for assay of the pro-drug had to be acidified post extraction with formic acid (to a final concentration of 0.1%). However, this procedure interfered with the assay of EDTA-diamide, where even small addition of formic acid to the sample significantly suppressed the MS signal. Therefore, after extraction all samples were divided into 2 aliquots where EDTA-diamide and ICRF-154 were analyzed in the first without acidification, and MST-16 was assayed in the second after addition of formic acid. The chromatograms of UHPLC-MS/MS analysis of the compounds in all tested biological materials are shown in Fig. 2.

The UHPLC-MS/MS method was validated for determination of all analytes in  the medium, the  buffer, rabbit plasma and NVCM cells according to EMA guideline^26^. The selectivity of the method was checked by comparing chromatograms of blank and quality control (QC) samples. Any significant coelution at the retention times of the analytes and I.S.s was detected (Fig. 2). The linearity, precision, accuracy and matrix effects are summarized for each matrix in Tables 1–4 and all were acceptable. Precision and accuracy were tested at 5 different concentrations for all media, with exception of the cells, where the number of samples was reduced to 4 due to decreased availability of the blank matrix. The precipitation led to recovery rates higher than 88 and 72% for all analytes in plasma and NVCMs, respectively, with acceptable reproducibility (Table 5). The post-preparative stability experiments proved sufficient stability of all analytes up to 10 hours, stored in an autosampler set at 15 °C (Table 5).

**Table 1:** Linearity, accuracy, precision and matrix factor for UHPLC-MS/MS assay of the analytes in the medium (DMEM).

Linearity								
Analyte	Range (µM)					r^2^		
MST-16	1–150					0.9993		
ICRF-154	1–150					0.9987		
EDTA-diamide	5–150					0.9993		
**Concentration added (µM)**	**Intra-day**			**Inter-day**			**MF**	
	**Precision**		**Accuracy**	**Precision**		**Accuracy**	**IS-normalized**	
	**Concentration found** ± **S.D. (µM)**	**R.S.D**.	**(%)**	**Concentration found** ± **S.D. (µM)**	**R.S.D**.	**(%)**	**(%)**	**R.S.D**.
**MST-16**								
1.0	1.1 ± 0.16	15.2	106.0	0.8 ± 0.07	9.1	80.2	97.9	3.3
3.0	3.1 ± 0.27	8.8	103.7	3.0 ± 0.13	4.2	100.1		
50.0	54.4 ± 2.86	5.3	108.9	43.5 ± 5.95	13.7	87.0		
100.0	101.9 ± 4.48	4.4	101.9	95.5 ± 4.04	4.2	95.5		
150.0	155.9 ± 12.97	12.5	103.9	153.8 ± 3.98	3.9	102.5	98.3	4.9
**ICRF-154**								
1.0	1.0 ± 0.04	4.1	103.1	1.0 ± 0.17	16.5	104.3	93.7	14.2
3.0	3.1 ± 0.10	3.2	103.8	2.8 ± 0.40	14.3	92.3		
50.0	52.5 ± 4.60	8.8	105.0	51.9 ± 2.09	4.0	103.9		
100.0	86.0 ± 7.98	9.3	86.0	99.5 ± 2.51	2.5	99.5		
150.0	132.8 ± 8.95	6.7	88.5	137.7 ± 10.65	7.7	91.8	106.0	2.5
**EDTA-diamide**								
5.0	5.2 ± 0.37	7.1	103.0	5.2 ± 0.48	9.2	105.3	108.3	6.6
10.0	9.2 ± 0.33	3.6	91.8	10.1 ± 0.61	6.0	100.7		
50.0	52.4 ± 2.99	5.7	104.9	53.5 ± 1.71	3.2	107.1		
100.0	93.4 ± 2.07	2.2	93.4	100.0 ± 1.59	1.6	100.0		
150.0	144.0 ± 7.57	5.3	96.0	154.3 ± 8.04	5.2	102.9	99.7	6.8

**Table 2:** Linearity, accuracy, precision and matrix factor for UHPLC-MS/MS assay of the analytes in  the buffer (ADS).

Linearity								
Analyte	Range (µM)					r^2^		
MST-16	1–150					0.9989		
ICRF-154	1–150					0.9969		
EDTA-diamide	5–150					0.9955		
**Concentration added (µM)**	**Intra-day**			**Inter-day**			**MF**	
	**Precision**		**Accuracy**	**Precision**		**Accuracy**	**IS-normalized**	
	**Concentration found** ± **S.D. (µM)**	**R.S.D**.	**(%)**	**Concentration found** ± **S.D. (µM)**	**R.S.D**.	**(%)**	**(%)**	**R.S.D**.
**MST-16**								
1.0	1.0 ± 0.08	7.9	97.2	0.9 ± 0.08	8.7	93.2	101.3	10.2
3.0	3.1 ± 0.14	4.5	102.3	3.2 ± 0.31	9.5	107.5		
50.0	54.0 ± 5.72	10.6	108.0	47.1 ± 5.36	11.4	94.1		
100.0	97.5 ± 4.90	5.0	97.5	94.9 ± 2.32	2.4	94.9		
150.0	154.2 ± 4.80	3.1	102.8	153.6 ± 10.68	7.0	102.4	102.7	2.9
**ICRF-154**								
1.0	1.1 ± 0.15	13.7	111.3	1.1 ± 0.16	15.2	107.5	99.2	7.6
3.0	3.3 ± 0.23	6.9	109.5	2.8 ± 0.24	8.5	94.4		
50.0	56.1 ± 4.94	8.8	112.2	56.5 ± 4.72	8.4	113.1		
100.0	104.7 ± 8.21	7.8	107.7	100.1 ± 14.46	14.4	100.1		
150.0	145.2 ± 13.04	9.0	96.8	161.5 ± 25.42	15.7	107.7	95.2	1.3
**EDTA-diamide**								
5.0	4.5 ± 0.30	6.7	90.1	4.9 ± 0.53	10.7	98.7	91.1	3.1
10.0	9.8 ± 0.41	4.2	97.6	9.0 ± 0.71	7.9	91.3		
50.0	48.5 ± 2.09	4.3	97.0	45.0 ± 5.51	12.3	90.0		
100.0	93.0 ± 0.68	0.7	93.0	104.1 ± 8.69	8.3	104.1		
150.0	137.4 ± 3.32	2.4	91.6	143.2 ± 8.20	5.7	95.4	112.6	1.3

**Table 3:** Linearity, accuracy, precision and matrix factor for UHPLC-MS/MS assay of the analytes in plasma.

Linearity								
Analyte	Range (µM)					r^2^		
MST-16	1–150					0.999		
ICRF-154	1–150					0.9963		
EDTA-diamide	5–150					0.9996		
**Concentration added (µM)**	**Intra-day**			**Inter-day**			**MF**	
	**Precision**		**Accuracy**	**Precision**		**Accuracy**	**IS-normalized**	
	**Concentration found** ± **S.D. (µM)**	**R.S.D**.	**(%)**	**Concentration found** ± **S.D. (µM)**	**R.S.D**.	**(%)**	**(%)**	**R.S.D**.
**MST-16**								
1.0	1.0 ± 0.05	5.2	100.4	1.0 ± 0.11	11.2	97.9	115.4	5.2
3.0	2.8 ± 0.18	6.7	91.6	2.9 ± 0.35	12.0	98.0		
50.0	51.4 ± 3.54	6.9	102.8	48.1 ± 6.79	14.1	96.3		
100.0	91.2 ± 5.14	5.6	91.2	96.3 ± 4.61	4.8	96.3		
150.0	148.8 ± 8.76	5.9	99.2	158.3 ± 12.05	7.6	105.5	106.7	2.5
**ICRF-154**								
1.0	1.2 ± 0.06	5.6	115.4	0.9 ± 0.10	10.6	94.1	105.4	8.3
3.0	2.7 ± 0.19	6.9	90.4	2.9 ± 0.42	14.3	97.8		
50.0	51.5 ± 5.75	11.2	103.1	43.0 ± 2.20	5.1	86.0		
100.0	98.8 ± 7.37	7.5	98.8	85.2 ± 5.97	7.00	85.2		
150.0	159.0 ± 7.24	4.6	106.0	137.5 ± 9.49	6.9	91.7	99.2	13.2
**EDTA-diamide**								
5.0	5.6 ± 0.33	5.9	111.8	5.5 ± 0.13	2.3	109.7	99.3	8.9
10.0	9.9 ± 0.30	3.0	99.1	9.8 ± 0.57	5.8	98.2		
50.0	49.2 ± 2.69	5.5	98.4	52.1 ± 4.78	9.2	104.3		
100.0	109.9 ± 3.52	3.2	109.9	99.7 ± 4.04	4.1	99.7		
150.0	161.9 ± 2.85	1.8	108.0	146.6 ± 6.50	4.4	97.7	105.3	2.7

**Table 4:** Linearity, accuracy, precision and matrix factor for UHPLC-MS/MS assay of the analytes in cardiac cells (NVCM).

Linearity									
Analyte	Range (pmol/10^6^ cells)					r^2^			
MST-16	5–100					0.9979			
ICRF-154	5–100					0.9989			
EDTA-diamide	5–100					0.9936			
**Concentration added (pmol/10** ^**6**^ **cells)**	**Intra-day**			**Inter-day**				**MF**	
	**Precision**		**Accuracy**	**Precision**			**Accuracy**	**IS-normalized**	
	**Concentration found** ± **S.D. (pmol/10**^**6**^ **cells)**	**R.S.D**.	**(%)**	**Concentration found** ± **S.D. (pmol/10**^**6**^ **cells)**	**R.S.D**.		**(%)**	**(%)**	**R.S.D**.
**MST-16**									
5.0	5.1 ± 0.13	2.5	102.2	5.1 ± 0.04	0.9		102.7	94.9	12.3
10.0	9.9 ± 0.26	2.6	99.4	9.0 ± 0.23	2.5		89.6		
50.0	47.2 ± 4.83	10.2	94.3	48.8 ± 6.04	12.4		97.7		
100.0	98.6 ± 2.77	2.8	98.6	98.2 ± 4.69	4.8		98.2	102.6	6.4
**ICRF-154**									
5.0	5.3 ± 0.23	4.4	105.6	5.1 ± 0.69	13.4		102.9	99.5	7.2
10.0	10.6 ± 0.04	0.4	106.4	9.4 ± 0.33	3.5		93.6		
50.0	50.6 ± 2.13	4.2	101.1	53.0 ± 4.98	9.4		106.0		
100.0	105.2 ± 2.94	2.8	105.2	102.9 ± 6.63	6.4		102.9	114.5	3.4
**EDTA-diamide**									
5.0	5.5 ± 0.34	6.2	110.3	5.3 ± 0.09	1.7		106.5	92.9	2.8
10.0	10.0 ± 0.56	5.6	100.5	9.7 ± 0.88	9.1		96.8		
50.0	49.6 ± 2.18	4.4	99.3	43.2 ± 4.67	10.8		86.3		
100.0	100.3 ± 12.08	12.0	100.3	89.4 ± 13.04	14.6		89.4	100.5	11.1

**Table 5:** Extraction recovery of the analytes from plasma and cardiac cells (NVCM) and post-preparative stability (10 hours in autosampler at 15 °C) of MST-16, ICRF-154 and EDTA-diamide in the medium (DMEM), the buffer (ADS), plasma and NVCM.

Extraction recovery				
Analyte	Plasma		NVCM	
	Recovery (%)	R.S.D.	Recovery (%)	R.S.D.
MST-16	95.3	9.0	100.6	8.4
ICRF-154	98.3	4.0	83.5	2.4
EDTA-diamide	88.9	11.1	72.3	5.7
**Post-preparative stability - % remaining**				
**Analyte**	**DMEM**	**ADS**	**Plasma**	**NVCM**
MST-16	96.0	96.6	105.6	107.5
ICRF-154	93.2	92.8	110.8	102.7
EDTA-diamide	100.5	106.7	98.2	96.5

Both extraction recovery and post-preparative stability were examined at the analytes’ concentrations of either 50 µM for DMEM, ADS buffer and plasma or 10 pmol/10^6^ cells for cardiac cells (NVCMs).

### Stability and bioactivation study of MST-16 in cell culture medium and cardiomyocytes

The UHPLC-MS/MS assay was utilized to examine the chemical stability of MST-16 in cell culture medium (and related buffer), metabolic stability in the presence of the cardiac cells in the medium, cellular penetration and the conversion of MST-16 to ICRF-154 and EDTA-diamide. The particular concentration (100 µM) used in these experiments was selected based on the relatively low toxicity of MST-16 towards cardiomyocytes found previously under similar conditions^16^. Furthermore, it is the same concentration as used previously with DEX without the signs of toxicity^27^. Regarding cytotoxicity of ICRF-154, it is generally known that bisdioxopiperazines show pronounced *in vitro* antiproliferative activity towards cancer cells^3^, but their toxicity is low or even negligible towards non-proliferating cells such as cardiomyocytes^16^. Hence, with respect to the conditions of the present experiments, low toxicity of ICRF-154 is expectable.

**Figure 3:**
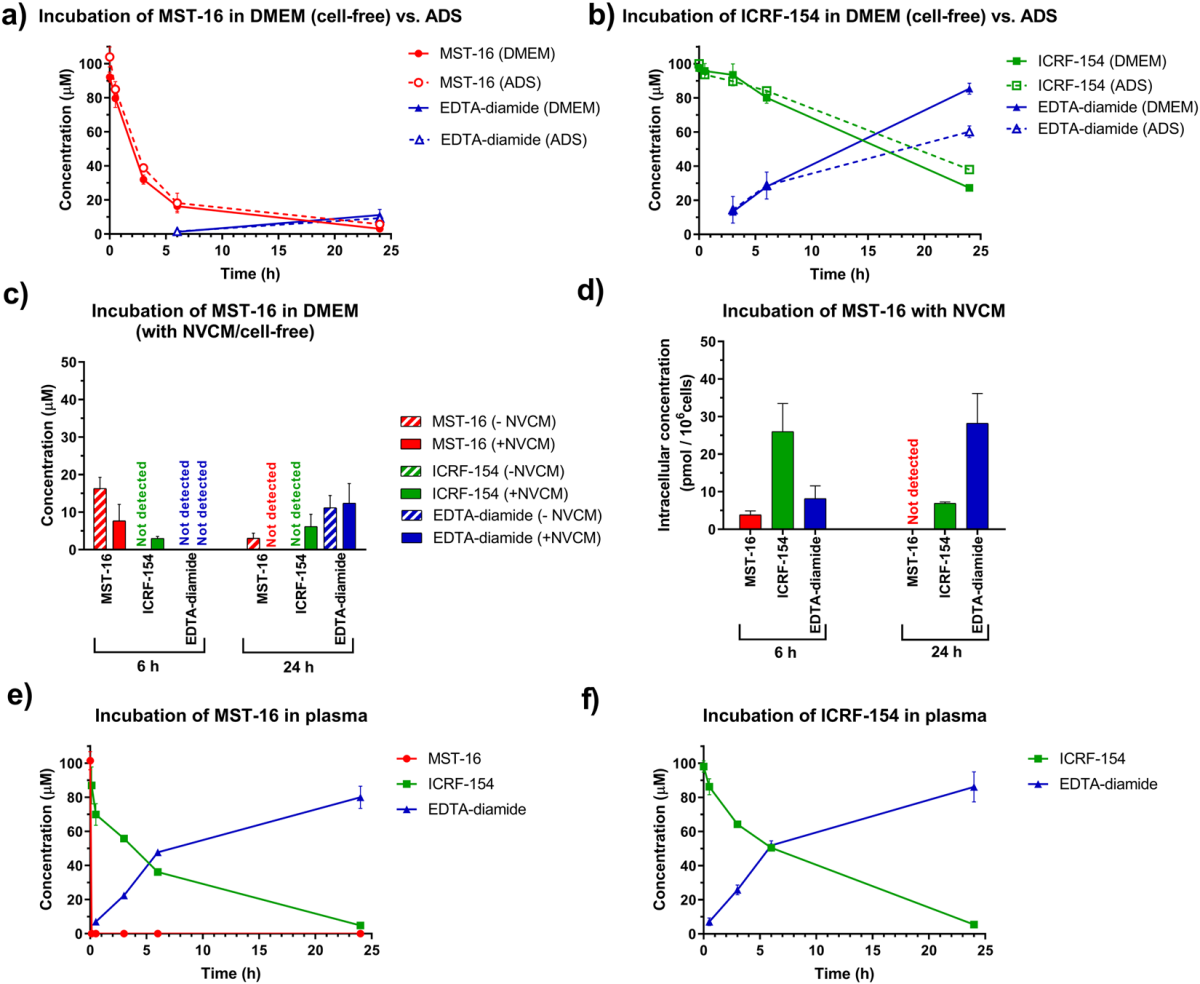
Concentration time profiles of the compounds assayed after incubation of **(a)** MST-16 or **(b)** ICRF-154 in the medium (DMEM) and the buffer  (ADS) and **(c)** MST-16 in DMEM with and without rat neonatal ventricular cardiomyocytes (NVCM); **(d)** intracellular concentrations of the compounds assayed after incubation of MST-16 with NVCM. Concentrations of the compounds assayed after incubation of **(e)** MST-16 or **(f)** ICRF-154 in rabbit plasma. All compounds were incubated at initial concentration of 100 µM at 37 °C. The results are expressed as the mean ± S.D. (n = 3).

When incubated in cell-free DMEM (pH 7.4), the concentrations of the pro-drug, MST-16, gradually declined to approximately 5% of the initial amount within 24 hours of the experiment (Fig. 3a). A similar trend in the degradation profile was observed when the pro-drug was incubated in the  related buffer (pH 7.4) (Fig. 3a). This indicates that the chemical degradation of MST-16 is not accelerated by specific components of the cell medium (e.g., amino acids, glucose, etc.), it is instead related to spontaneous hydrolysis. The concentrations of ICRF-154 assayed after MST-16 incubation were below lower limit of quantification (LLOQ) of the method (i.e., 1 µM) during the entire experiment and only 10 µM of EDTA-diamide were found at the end of the study (24 hours). The concentrations of these degradation products did not correspond with the rate of decomposition of the pro-drug which could be related to the presence of other unrecognized degradation products. Hydroxymethyl-ICRF-154, that has been proposed as an intermediate of MST-16 activation by Swift *et al*.^4^, was found unstable under conditions of UHPLC-MS/MS analysis. Therefore, hydroxymethyl-ICRF-154 originating from MST-16 degradation is presumably assayed in this study as ICRF-154. This observation is in agreement with the rapid release of formaldehyde from hydroxymethyl-ICRF-154 that was reported previously^4^. The other unrecognized degradation product may be an intermediate of the bisdioxopiperazine rings’ opening, analogically as in DEX^9,28^. However, we did not detect this potential degradation product in the incubated samples.

Other explanation of the low mass balance may be an extremely poor solubility of ICRF-154 that originated from MST-16 in these aqueous and protein-free environments. The low solubility of ICRF-154 need not be an issue in plasma which is known for marked solubilizing properties.

When ICRF-154 (100 µM) was incubated alone in the same media, the compound gradually degraded to EDTA-diamide (Fig. 3b) and no precipitation was observed. Similar to MST-16, the components of the cell medium had no significant effect on the stability of ICRF-154 in most time intervals tested (Fig. 3b).

The concentrations of MST-16, ICRF-154 and EDTA-diamide were determined both in the cell medium and inside the NVCMs after 6 and 24 hours incubation with the pro-drug. The selected time periods represent relevant times of the well-established model for study of anthracycline-induced cardiotoxicity *in vitro*. As apparent in Fig. 3c, NVCM cells accelerated the decrease in the concentration of MST-16 in the cell medium, which implies metabolic activity of the cardiomyocytes. Unlike in the cell-free medium, approximately 3 µM and 6 µM concentrations of ICRF-154 were determined in the medium with NVCMs after 6 and 24 hours of the incubation, respectively. EDTA-diamide was determined at similar concentrations (approximately 12 µM) in the media both with and without NVCMs after 24 hours. The intact pro-drug, its active form, ICRF-154, as well as its metabolite, EDTA-diamide, could be detected inside NVCM cells after 6 hours of incubation with MST-16 (Fig. 3d). However, unlike in the cell medium where MST-16 was the prevalent compound, intracellular concentrations of the pro-drug were lower (close to LLOQ, 5 pmol/10^6^ cells) compared to its active form (27 pmol/10^6^ cells). Supposing that ICRF-154 does not penetrate into the cells faster than the more lipophilic pro-drug, the intracellular content of ICRF-154 should be a product of activation of MST-16 inside the cells. After 24 hours incubation, MST-16 was bellow LLOQ and ICRF-154 content fell to 7 pmol/10^6^ cells, while EDTA-diamide was the prevailing form of the drug (Fig. 3d). ICRF-154 and other bisdioxopiperazines have been reported to enter the cells by passive diffusion^29,30^. No such data are available for the pro-drug MST-16. Contrary to ICRF-154, penetration of EDTA-diamide into cardiomyocytes may require an active membrane transport, analogically to what we have previously described in ADR-925^27^. However, the particular transporter(s) responsible for this process have not been identified yet^27^. Of note, while transport of ADR-925 from the cell culture media into the cells may indeed contribute to the intracellular concentrations of this metabolite, the concentrations of EDTA-diamide determined in the present study in the cell medium (Fig. 3c) do not suggest that it should be the major determinant of its relatively high intracellular content (the concentrations in the media were lower than 5 and 12 µM at 6 and 24 hours of the experiment, respectively). Hence, the majority of EDTA-diamide detected in this study inside NVCMs probably originated from metabolism of ICRF-154.

Esterases, that are ubiquitous in various biological systems including rat cardiac cells^31^, may be involved in conversion of MST-16 to ICRF-154^4^. By analogy to DEX, ICRF-154 could be initially metabolized to the single-ring open intermediate by catalysis of dihydropyrimidase (DHPase) and then the intermediate could be hydrolyzed to EDTA-diamide with contribution of dihydroorotase (DHOase)^32,33^. However, it is unlikely that these enzymes are responsible for metabolism of ICRF-154 to EDTA-diamide in cardiac cells, as DHPase was not found in the heart^30,32,34^ and DHOase has no hydrolyzing activity directly on DEX^33^. Furthermore, we and others^27,32^ have previously shown that lysates of cardiomyocytes and myocardium do not significantly accelerate hydrolytic opening of DEX which contrasted with the lysates from other organs.

### Stability and bioactivation study of MST-16 in rabbit plasma

The concentration profiles of MST-16, ICRF-154 and EDTA-diamide were assayed in rabbit plasma after incubation at a physiologically relevant temperature (37 °C) *in vitro* with either MST-16 (Fig. 3e) or ICRF-154 (Fig. 3f). MST-16 was immediately decomposed and after 5 minutes of the incubation, the concentration of the pro-drug fell below 1 µM. The rapid increase in ICRF-154 was followed by its metabolism to EDTA-diamide (Fig. 3e). Both ICRF-154 and EDTA-diamide were found in an approximately equimolar ratio at 6 hours incubation, while EDTA-diamide was the major form of the drug present in plasma after 24 hours. The analogical profile of ICRF-154 and EDTA-diamide concentrations was observed when ICRF-154 alone was incubated in plasma under the same conditions (Fig. 3f). The faster degradation of both MST-16 and ICRF-154 in plasma as compared to the cell culture medium and the buffer of the same pH, implied that some plasma components accelerated this process. Indeed, enzymatic catalysis by plasma esterases may be involved in fast conversion of MST-16 to ICRF-154 in plasma^4^. Nevertheless, DHPase and DHOase are unlikely to be present in plasma, thus ICRF-154 hydrolysis to EDTA-diamide is likely promoted by a non-enzymatic process (e.g. with contribution of metal ions). The acceleration of the hydrolysis rate of DEX in the presence of ions was previously observed by others^35,36^. This is also in agreement with our recent study where DEX degradation in plasma under the same conditions was faster compared to the cell culture media and the corresponding buffer^27^.

Although ICRF-154 and EDTA-diamide were the main products originating from MST-16 in plasma, the mass balance calculations particularly for the early time intervals of the experiment suggested the presence of other undetected products. We found that hydroxymethyl-ICRF-154 was unstable and was detected as ICRF-154. Furthermore, the single-ring opened intermediate product of ICRF-154 metabolism to EDTA-diamide may also be responsible for this phenomenon, analogically to what was described for DEX^9,37^. Fast conversion of MST-16 to ICRF-154 in plasma is in agreement with a previous study, where only ICRF-154 was determined in plasma after administration of the pro-drug to rats^5^.

## Conclusions

This study presents the first validated UHPLC-MS/MS assay of the lipophilic pro-drug sobuzoxane (MST-16), along with its active form, bisdioxopiperazine (ICRF-154), and the polar metabolite (EDTA-diamide). This assay was applied to the analysis of the biological samples taken from *in vitro* experiments aimed at examination of stability, bioactivation and cellular penetration of MST-16. We showed that MST-16 gradually decomposed in all tested media, but this process was particularly accelerated in plasma, where ICRF-154 and EDTA-diamide were the main degradation products. However, minor amount of the intermediate was also likely present in the incubated samples. Cardiac cells had a metabolic activity towards MST-16 and accelerated its activation to ICRF-154 in the cell culture medium. The pro-drug is metabolized inside cardiac cells to ICRF-154, and then to EDTA-diamide, the latter being the predominant form of the drug detected inside the cells after 24 hours of incubation. Hence, this study presents a modern analytical tool for the investigation of MST-16 activation and provides initial data on the fate of the pro-drug under *in vitro* conditions relevant to examination of its cardioprotective effects.

## Methods

### Chemicals and materials

Sobuzoxane (MST-16, MW 514), hydroxymethyl-ICRF-154 (MW 314), ICRF-154 (MW 254), EDTA-diamide (MW 290) and the internal standards (Fig. 1b), I.S._MST-16_ (MW 486), I.S._ICRF-154_ (racemic form of DEX, MW 268) and I.S._EDTA-diamide_ (MW 304) were synthesized in-house as described in the Supplementary Material. All substances were characterized by NMR and MS (see Supplementary Material). Blank rabbit plasma (with heparin as an anti-coagulant) from healthy rabbits was either purchased from commercial source (Zooservis a.s., Czech Republic) or provided by the Faculty of Medicine in Hradec Kralove from *in vivo* experiments approved by Animal Welfare Committee of the Faculty of Medicine in Hradec Kralove, Charles University (Czech Republic) and conforming to the Guide for the Care and Use of Laboratory Animals^38^.

Dulbecco’s Modified Eagle’s medium containing the nutrient mixture F-12 (DMEM/F12), penicillin/streptomycin solution (5000 U/mL; P/S) and sodium pyruvate solution (100 mM; PYR) were purchased from Lonza (Switzerland). Horse serum (HS), fetal bovine serum (FBS), 4-(2-hydroxyethyl)-1-piperazineethanesulfonic acid (HEPES) and phosphate buffered saline (PBS) tablets were purchased from Sigma-Aldrich (Germany).

The sera were heat-inactivated prior to use (56 °C, 30 minutes). Neonatal ventricular rat cardiomyocytes were isolated, as described below (section “Cell culture experiment”). Other chemicals and solvents used for chromatographic analysis, sample preparation and *in vitro* experiments were purchased from Sigma-Aldrich (Germany) and were of either chromatographic or MS purity. Milli-Q water was prepared using a Millipore purification system (Merck-Millipore, Germany). Millex-GV filters (PVDF, 0.22 μm) for filtration of samples before analysis were purchased from Sigma-Aldrich (Germany). The buffered saline of pH 7.4 (ADS buffer) was prepared in house using Millipore water supplemented with 116 mM NaCl, 5.3 mM KCl, 1 mM CaCl_2_, 1.2 mM MgSO_4_, 1.13 mM NaH_2_PO_4_, 5 mM glucose, and 20 mM HEPES (all components were from Penta, Czech Republic or Sigma-Aldrich, Germany).

### Stock and working solutions

The stock solutions (concentrations of 25 mM for MST-16, 10 mM for ICRF-154, 1 mM for EDTA-diamide and 4 mM for all I.S.) were prepared by dissolving an appropriate amount of the substance in the following solvents: DMSO (MST-16 and ICRF-154), acetonitrile (I.S._MST-16_), methanol (I.S._ICRF-154_) and a mixture of methanol and water (1:1, *v/v*) (EDTA-diamide, I.S._EDTA-diamide_).The stock solutions were stored for up to one month at 4 °C. The stock solutions were diluted with the same solvent to get a set of working solutions of different concentrations for each analyte (25–4000 µM for MST-16 and ICRF-154, 6–900 µM for EDTA-diamide) and two concentrations of all I.S. (150 and 1000 µM). The working solutions were stored for up to 7 days at 4 °C.

### Chromatographic analysis

#### UHPLC-MS/MS  method

Analyses were performed using a NexeraX2 UHPLC system coupled to a LCMS-8030 triple quadrupole mass spectrometer (both Shimadzu, Japan) with electrospray ionization source (ESI) working in a positive ion mode. The acquired data were processed using LabSolutions software (v. 5.60 SP2, 2013, Shimadzu, Japan). The following columns were tested in method development: Synergi 4 µm Polar-RP (3 × 150 mm or 75 mm, 4 µm; Phenomenex, USA), Hypercarb (3 × 100 mm, 3 µm, Thermo Scientific, USA) and Zorbax SB-Aq (3 × 150 mm, 3.5 µm or 3 × 100 mm, 1.8 µm, Agilent, USA), in combination with different mobile phases. Water, 0.1% formic acid or 0.5–2 mM ammonium formate and methanol or acetonitrile were tested as mobile phases A and B, respectively, in different gradients. The optimal separation was achieved on Zorbax SB-Aq column (3 × 100 mm, 1.8 µm, Agilent, USA) employing 1 mM ammonium formate (A) and methanol (B) in the following gradient: 0.0–1.5 minutes (5% B), 1.5–5.0 minutes (5–90% B), 5.0–8.5 minutes (90% B), 8.50–13.00 minutes (5% B). The column, along with the guard column, were flushed with a mixture of 2 mM ethylenediaminetetraacetic acid dipotassium (K_2_EDTA) aqueous solution and acetonitrile (90:10, *v/v*) prior to the first analysis (3 hours using a flow rate of 0.3 ml/minutes). This step was necessary to minimize loss of sensitivity for the chelating analytes due to the formation of complexes with the trace amount of metals present in the chromatographic system^23^. The flow rate of 0.3 ml/minutes, column temperature of 30 °C and autosampler temperature of 15 °C were used. Three µl of each sample were injected onto the column. Structural analogs of the analytes were used as internal standards (Fig. 1b). The mass spectrometer was tuned automatically for all analytes and internal standards. The following setup was used: nebulizing gas flow rate of 3 l/min, drying gas flow rate of 15 l/min, interface voltage of 4.5 kV, DL temperature of 250 °C, heat block temperature of 400 °C, CID gas pressure 230 kPa. Quantitation was done using selected reaction monitoring (SRM) with unit mass resolution and a dwell time of 5 ms. The run was divided into 3 acquisition time segments. Selected reaction monitoring conditions were tuned for each segment separately to fit for monitoring of the particular analyte and corresponding internal standard. The most abundant precursors corresponded mainly to protonated molecule [M + H]^+^, except for MST-16 and I.S._MST-16_ (all media) and EDTA-diamide and I.S. _EDTA-diamide_ (NVCM), where adduct ion with either ammonium [M + NH_4_]^+^ or sodium [M + Na]^+^ was used, respectively. The details on MS settings are summarized in Table 6.

**Table 6:** The details on SRM used for quantitation of the analytes. *Each SRM transition was repeated 5×; **SRM conditions used for assay of EDTA-diamide and I.S._EDTA-diamide_ in cardiac cell samples (NVCM).

Compound	Retention time (min)	Acquisition segment (min)	Precursor ion* *(m/z)*	Product ion *(m/z)*	Collision energy (eV)
EDTA-diamide	1.4	0.8–2.1	290.9	159.0	−20
			312.8**	255.1**	−24**
I.S_EDTA-diamide_	1.5	0.8–2.1	305.1	173.1	−14
			327.1**	195.1**	−30**
ICRF-154	4.8	4.0–6.3	255.2	141.1	−15
I.S_ICRF-154_	5.2	4.0–6.3	269.1	155.2	−14
I.S_MST-16_	6.7	6.0–7.5	504.0	383.1	−14
MST-16	6.9	6.0–7.5	532.3	341.1	−20

#### Sample preparation

Fifty microliters of DMEM medium or ADS buffer were diluted 20× with either an ice-cold mixture of methanol and water (20:80, *v/v*) or the same mixture with addition of 0.1% formic acid. Fifty microliters of plasma were precipitated on ice with 300 μl of ice-cold methanol or methanol with the addition of 0.1% formic acid. The samples were vigorously mixed for 30 seconds on a Vortex and then centrifuged (10 minutes, 10,000 rpm, 4 °C). The supernatant was then filtered (PVDF, 0.22 µm). While EDTA-diamide and ICRF-154 were assayed in samples without 0.1% formic acid, MST-16 had to be quantified in acidified samples to improve its post-preparative stability.

NVCM pellets (4.8 × 10^6^ cells) were homogenized in an ultrasonic bath (2 minutes) and ice-cold methanol (300 µl) was added. The samples were vigorously mixed for 30 seconds on a Vortex and then centrifuged (10 minutes, 10,000 rpm, 4 °C). The supernatant was then filtered (PVDF, 0.22 µm) and divided into two aliquots. The first one was analyzed immediately to determine EDTA-diamide and ICRF-154 while the second one was acidified with formic acid (to achieve a final concentration of 0.1%) for assay of MST-16.

#### Method validation

The method was validated for determination of MST-16, ICRF-154 and EDTA-diamide in DMEM medium, ADS buffer, rabbit plasma and cardiac  cell samples according to EMA Guideline on Bioanalytical Method Validation^26^. Blank DMEM medium, ADS buffer, rabbit plasma and NVCM cells (4.8 × 10^6^ per pellet) were used to prepare quality control (QC) samples for validation purposes. Linearity was tested within the concentration range from either 1 or 5 µM to 150 µM, for MST-16, ICRF-154 or EDTA-diamide, respectively, in DMEM medium, ADS buffer and plasma and from 5 to 100 pmol/10^6^ cells for all compounds in NVCM samples. The lowest concentrations represent the lower limit of quantification(LLOQ). Accuracy and precision were evaluated at either 4 (cell samples) or 5 (DMEM, ADS, and plasma) different concentration levels covering LLOQ, low, medium and high concentrations and using at least 5 QC samples at each level; they were expressed as the mean percentage of the determined amount relative to spiked amount of the substance and R.S.D. (%) of analyses after repeated sample preparation, respectively. Selectivity was checked by examination of the detector response from analysis of blank biological materials at the retention times of the analytes. The matrix effect was investigated using plasma taken from different animals, cardiac cells from different isolations and DMEM medium and ADS buffer from different lots. The matrix effect was tested at low and high concentrations (n = 5 at each level) and was calculated by comparing the detector response obtained from analysis of the standards and the post-extraction spiked biological samples; it was expressed as the mean I.S.-normalized matrix factor (%) and corresponding R.S.D (%). Recovery of precipitation of the analytes from plasma and NVCM cells was determined at the medium concentration level (n = 5) and was calculated by comparing the detector response for analysis of the spiked biological sample and the biological sample spiked post precipitation. Post-preparation stability was tested during 10 hours in an autosampler (15 °C) using the medium concentration of the analyte (n = 3, per biological material). All biological samples were treated and analyzed immediately after collection to prevent artificial conversion of MST-16  to ICRF-154, thus the freeze-thaw stability did not need to be examined.

### *In vitro* stability experiments in DMEM medium, ADS buffer and plasma

DMEM medium, ADS buffer (both pH 7.4) or rabbit plasma was incubated with either MST-16 (100 µM) or ICRF-154 (100 µM) at 37 °C for 24 hours (under gentle mixing, 350 rpm). They were sampled at 0, 0.5, 1, 3, 6, and 24 hours for DMEM medium  and ADS buffer or 0, 5 minutes, 0.5, 1, 3, 6 and 24 hours for rabbit plasma. The samples were treated as described above (section “Sample preparation”) and immediately analyzed. The experiments were done in triplicate and the results were expressed as the mean ± S.D.

### Cell culture experiment

The use of experimental animals for primary cell culture isolation has been approved by Charles University, Faculty of Pharmacy Animal Welfare Committee. All experiments were performed in accordance with Directive 2010/63/EU on the Protection of Animals Used for Scientific Purposes. Neonatal ventricular rat cardiomyocytes were isolated from 1–3-day-old Wistar rats as described previously^39^. Briefly, neonatal hearts were minced in ADS buffer on ice, digested at 37 °C with type II collagenase (Invitrogen, USA) and pancreatin (Sigma-Aldrich, Germany). After preplating on 150 mm Petri dishes (approximately 20 hearts on each dish with 2 hours of incubation) to minimize nonmyocyte contamination, the  cardiomyocytes were plated on 60 mm Petri dishes, precoated with gelatin at a density of 4.8 × 10^6^  cells per dish. The  cardiomyocytes were cultured at 37 °C and 5% CO_2_ in DMEM/F12 supplemented with 10% HS, 5% FBS, 20% PYR and 1% penicillin/streptomycin solution. Newly isolated  cardiomyocytes had been left for 40 hours to attach properly and form a monolayer of spontaneously beating cardiomyocytes, then the culture medium was changed to serum-free DMEM/F12, in which the  cardiomyocytes were maintained to the end of the experiment. The cardiomyocytes were incubated with MST-16 (100 µM) for 24 hours. At selected time intervals (6 and 24 hours) samples of the cell culture media were collected for analysis and the  cardiomyocytes were washed twice with PBS buffer at 4 °C, harvested using a cell scraper into a small volume of PBS and the solution was centrifuged (700 × g, 10 minutes, 4 °C). The supernatant was discarded and the pellet was immediately treated as described in the section “Sample preparation” and analyzed. All experiments were done in triplicate and the results were expressed as the mean ± S.D.

## Supplementary information

Supplementary information

## Data Availability

All data generated or analyzed during this study are included in this published article (and its Supplementary Information file).
