# Supplementary material for: UHPLC-MS/MS method for analysis of sobuzoxane, its active form ICRF-154 and metabolite EDTA-diamide and its application to bioactivation study
Source: Sci Rep. 2019 Mar 14;9:4524. doi: 10.1038/s41598-019-40928-5 (PMC6418109; doi:10.1038/s41598-019-40928-5)
Supplement: Supplementary file 1 — Supplementary information [file 41598_2019_40928_MOESM1_ESM.docx]

**Supplementary Information**

**UHPLC-MS/MS method for analysis of sobuzoxane, its active form ICRF-154 and metabolite EDTA-diamide and its application to bioactivation study**

Petra Reimerová, Anna Jirkovská, Hana Bavlovič Piskáčková, Galina Karabanovich, Jaroslav Roh, Tomáš Šimůnek, Petra Štěrbová-Kovaříková*

**Chemistry**

**General.**

Prepared compounds were characterized using ^1^H-NMR and ^13^C-NMR spectroscopy. ^1^H and ^13^C NMR spectra were recorded with Varian Marcury Vx BB 300 or VNMR S500 NMR spectrometers (Varian, Palo Alto, CA, USA). Chemical shifts were reported as δ values in parts per million (ppm) and were indirectly referenced to tetramethylsilane (TMS) via the solvent signal. All chemicals were purchased from Sigma-Aldrich (Germany) and were used without further purification. TLC was performed on Merck aluminum plates with silica gel 60 F_254_. Merck Kieselgel 60 (0.040-0.063 mm) was used for column chromatography.

**Synthesis of sobuzoxane (MST-16) and I.S._MST-16_.**

Sobuzoxane and its propyl homologue were prepared according to previously published procedure.[1]

Preparation of 4,4'-(ethane-1,2-diyl)bis(1-(hydroxymethyl)piperazine-2,6-dione) (**hydroxymethyl-ICFR-154**)

The suspension of 4,4’-(ethane-1,2-diyl)bis(piperazine-2,6-dione) (5 g, 0.02 mol) in DMF (10 mL) was heated at 130 °C for 10 min followed by the addition of 37 % formaldehyde (5.4 mL, 0.068 mol). The suspension turned into a clear solution. The reaction mixture was heated at 130 °C for 1.5 hours. Upon completion, the reaction mixture was cooled down and 50 mL of mixture of Et_2_O and EtOAc (2:1) was added. The precipitated product was filtered, washed with Et_2_O (10 mL) and dried. Yield: 70% (4.31 g) of 4,4`-(ethane-1,2-diyl)bis((1-hydroxymethyl)piperazine-2,6-dion) as a white solid.^1^H NMR (500 MHz, DMSO-*d*_6_) δ 6.17 (s, 2H, O*H*), 5.02 (s, 4H), 3.48 (s, 8H), 2.61 (s, 4H). ^13^C NMR (126 MHz, DMSO-*d*_6_) δ 170.22, 61.14, 55.81, 51.81.

Preparation of 4,4'-(1,2-ethanediyl)bis(1-(isobutyloxycarbonyloxymethyl)piperazine-2,6-dione) (sobuzoxane, **MST-16**):

Isobutyl chloroformate (2.6 mL, 0.02 mol) was added dropwise to a suspension of 4,4'-(ethane-1,2-diyl)bis(1-(hydroxymethyl)piperazine-2,6-dione) (3 g, 0.0095 mol) in pyridine (30 mL) under argon atmosphere at 0 °C. The reaction mixture was stirred at rt overnight. The solvent was evaporated under reduced pressure and the crude product was dissolved in CHCl_3_ (50 mL). The resulting solution was washed with 10% aq. HCl (1 × 50 mL) and with water (2 × 30 mL). Organic phase was separated, dried over anhydrous Na_2_SO_4_, and evaporated under reduced pressure. The product was purified using column chromatography (mobile phase: CDCl_3_). Yield: 53% (2.55 g) of sobuzoxane (MST-16) as a white solid. ^1^H NMR (500 MHz, CDCl_3_) δ 5.82 (s, 4H), 3.95 (d, *J* = 6.7 Hz, 4H), 3.53 (s, 8H), 2.68 (s, 4H), 2.01-1.93 (m, 2H), 0.94 (d, *J* = 6.7 Hz, 12H). ^13^C NMR (126 MHz, CDCl_3_) δ 168.38, 153.66, 74.60, 64.11, 56.34, 52.82, 27.68, 18.82.

Preparation of 4,4'-(1,2-ethanediyl)bis(1-(propoxycarbonyloxymethyl)piperazine-2,6-dione) (**I.S._MST-16_**)

Propyl chloroformate (0.21 mL, 1.91 mmol) was added dropwise to a suspension of 4,4'-(ethane-1,2-diyl)bis(1-(hydroxymethyl)piperazine-2,6-dione) (0.3 g, 0.95 mmol) in pyridine (3 mL) under argon atmosphere at 0 °C. The reaction mixture was stirred at rt overnight. The solvent was evaporated under reduced pressure and the crude product was dissolved in CHCl_3_ (20 mL). The resulting solution was washed with 10% aq. HCl (1 × 15 mL) and with water (2 × 20 mL). Organic phase was separated, dried over anhydrous Na_2_SO_4_, and evaporated under reduced pressure. The product (I.S._MST-16_) was purified using column chromatography (mobile phase: CHCl_3_). Yield: 62% (0.3 g). ^1^H NMR (500 MHz, CDCl_3_) δ 5.82 (s, 4H), 4.13 (t, *J* = 6.7 Hz, 4H), 3.53 (s, 8H), 2.68 (s, 4H), 1.76 – 1.63 (m, 4H), 1.00 – 0.90 (m, 6H). ^13^C NMR (126 MHz, CDCl_3_) δ 168.41, 153.60, 70.17, 64.09, 56.33, 52.80, 21.87, 10.10.

**Synthesis of razoxane (I.S._ICRF-154_)**.

Razoxane, the racemic form of the cardioprotective drug dexrazoxane, was prepared according to previously published procedure.[2]

1,2-Diaminopropane-*N*,*N*,*N*′,*N*′-tetraacetic acid (31.5 g, 0.103 mol) was mixed with formamide (126 mL) and the reaction mixture was heated under reduced pressure (30 mbar) at 110 °C for 1.5 hour. The suspension turned into the uncolored solution. Then, the reaction vessel was filled with argon and the reaction mixture was heated for 5 hours at 150-160 °C under argon atmosphere. Upon completion, formamide was distilled off under reduced pressure (30 mbar), the residue was cooled to rt and MeOH (50 mL) was added. The resulting suspension was stirred at rt overnight. White crystalline product was filtered, washed with another 50 mL of MeOH and dried. Yield: 57% (15.8 g) of razoxane (I.S._ICRF-154_) as a white solid. ^1^H NMR (300 MHz, DMSO-*d*_6_) δ 11.07 (s, 1H), 11.00 (s, 1H), 3.37 – 3.30 (m, 8H), 3.09 – 2.98 (m, 1H), 2.58 (dd, *J* = 13.0, 7.8 Hz, 1H), 2.31 (dd, *J* = 13.0, 6.1 Hz, 1H), 0.88 (d, *J* = 6.5 Hz, 3H). ^13^C NMR (75 MHz, DMSO-*d*_6_) δ 172.22, 171.69, 57.71, 55.38, 53.64, 51.54, 12.77.

**Synthesis of ICRF‑154**

4,4’-(Ethane-1,2-diyl)bis(piperazine-2,6-dion) (ICRF-154) was prepared using the same protocol as for the preparation of razoxane. Ethylenediaminetetraacetic acid (EDTA) (40 g, 0.137 mol) was used as a starting material. Yield: 66% (22.94 g) of 4,4’-(ethane-1,2-diyl)bis(piperazine-2,6-dione) (ICRF-154) as a white solid. ^1^H NMR (500 MHz, DMSO-*d*_6_) δ 11.07 (s, 2H), 3.34 (s, 8H), 2.59 (s, 4H). ^13^C NMR (126 MHz, DMSO-*d*_6_) δ 171.56, 55.21, 52.05.

**Synthesis of the racemic form of ADR-925 (I.S._EDTA-diamide_)**

The racemic form of ADR-925 (I.S._EDTA-diamide_) was prepared as described previously.[3]

The solution of razoxane (10 g, 37.3 mmol) in 0.5M aqueous NaOH (149.2 mL, 74.6 mmol) was stirred at rt for 24 hours. Upon completion, as determined by TLC (mobile phase: CHCl_3_/CH_3_OH, 2:1; detection with iodine vapours), the reaction mixture was acidified with Amberlyst^®^ 15 (hydrogen form) to pH 5-6. Amberlyst 15 was filtered off and the clear aqueous filtrate was evaporated to dryness under reduced pressure. Product was further dried under reduced pressure over P_2_O_5_. Yield: 92% (11g) of the racemic form of ADR-925 monohydrate (I.S._EDTA-diamide_). ^1^H NMR (500 MHz, D_2_O) δ 3.90 – 3.73 (m, 4H), 3.68 – 3.49 (m, 5H), 3.23 (dd, *J* = 14.7, 3.9 Hz, 1H), 3.00 (dd, *J* = 14.6, 11.7 Hz, 1H), 1.19 (d, *J* = 6.6 Hz, 3H). ^13^C NMR (126 MHz, D_2_O) δ 174.59, 173.84, 173.51, 171.59, 57.10, 56.76, 56.62, 56.42, 53.68, 53.59, 10.60. Anal. Calcd for C_11_H_22_N_4_O_7_: C, 40.99; H, 6.88; N, 17.38. Found: C, 40.52; H, 6.62; N, 17.1.

**Synthesis of EDTA-diamide**

*N*,*N*'-bis(carbamoylmethyl)ethylenediamine-*N*,*N*'-diacetic acid was prepared analogously. The reaction of 4,4’-(ethane-1,2-diyl)bis(piperazine-2,6-dion) (1 g, 3.93 mmol) with 1 M NaOH (7.87 mL, 7.87 mmol) resulted in 0.37g (32%) of *N*,*N*'-bis(carbamoylmethyl)ethylenediamine-*N*,*N*'-diacetic acid (EDTA-diamide). ^1^H NMR (500 MHz, D_2_O) δ 3.78 (s, 4H), 3.68 (s, 4H), 3.25 (s, 4H). ^13^C NMR (126 MHz, D_2_O) δ 171.94, 171.42, 55.98, 55.89, 51.28.

**References**

1. Cai JC, Shu HL, Tang CF, Komatsu T, Matsuno T, Narita T, et al. Synthesis and antitumor properties of N-1-acyloxymethyl derivatives of bis(2,6-dioxopiperazines). Chem Pharm Bull. 1989;37(11):2976-83.

2. Creighton AM, inventorPatent US3941790: Bis diketopiperazines patent US3941790. 1976.

3. Jirkovsky E, Jirkovska A, Bures J, Chladek J, Lencova O, Stariat J, et al. Pharmacokinetics of the cardioprotective drug dexrazoxane and its active metabolite ADR-925 with focus on cardiomyocytes and the heart. J Pharmacol Exp Ther. 2018;364(3):433-46.
